# Differences between congenital-syphilis presenting as sepsis and neonatal sepsis: A case-control study

**DOI:** 10.1097/MD.0000000000017744

**Published:** 2019-11-01

**Authors:** Yang Liu, Yu Zhu, Yibin Wang, Chaomin Wan

**Affiliations:** a Department of Pediatrics, West China Second Hospital, Sichuan University; b Key Laboratory of Birth Defects and Related Diseases of Women and Children, Ministry of Education, Chengdu, People's Republic of China.

**Keywords:** clinical feature, congenital syphilis, neonatal sepsis

## Abstract

Congenital syphilis (CS) can cause serious impact on the fetus. However, congenital syphilis presenting as sepsis is a critical condition but hardly identified by the clinic for the first time. In this study, we aimed to identify the benefit of earlier and accurate diagnosis for the infants who suffer congenital syphilis presenting as sepsis.

A retrospective study was performed with patients diagnosed of congenital syphilis presenting as sepsis who were the inpatients in the West China Second Hospital between 2011 and 2018. The control group was collected in the neonatal sepsis patients whose blood culture are positive.

Fifty-eight patients were included in the study. In the congenital syphilis group, one patient died and 12 (41.3%) patients get worse to MODS (multiple organ dysfunction syndrome). Symptoms, signs, and lab examinations are found to be significantly different (*P* < .05) between two groups as below, including rash, palmoplantar desquamation, abdominal distension, splenomegaly, hepatomegaly, etc. And, at the aspect of Hb, PLT, WBC, CRP, ALT, AST, these differences occurred in the different groups. It is obvious that the prognosis of children with syphilis is worse. According to a comparison between the different outcomes in the CS, the worse outcome subgroup of patients is significantly younger and have more severely impaired liver function.

Because of the high mortality of these infants, pediatricians should improve awareness of CS. Syphilis screening is recommended for pregnant women.

## 1 Introduction

Congenital syphilis (CS), the result of fetal infection with *Treponema pallidum*, has become a health issue for a long time.^[1]^ It can cause serious impacts on the fetus at the stages of growth, development, and organogenesis. Infants can become infected via transplacental transmission from an infected mother as early as 9–10 weeks of gestation,^[2]^ which may have pathological changes during the pregnancy period. The most affected systems and organs are the skeletal, brain, liver, and lung.^[3]^ These infants may be asymptomatic or have clinical manifestations due to multiple organ involvement, such as hepatosplenomegaly, sepsis, and meningitis.^[4]^

Despite a wide understanding of the disease and preventive strategies, congenital syphilis remains a major public health problem globally, due to its high burden of morbidity and mortality.^[5]^ In 2012, an estimated 350,000 adverse pregnancy outcomes worldwide were attributed to syphilis, including 143,000 early fetal deaths/stillbirths, 62,000 neonatal deaths, 44,000 preterm/low-birth-weight babies, and 102,000 infected infants.^[6]^

However, diagnosis and management of CS may be confusing as more than half of infants are asymptomatic, signs in symptomatic infants may be subtle and nonspecific,^[7]^ and misinformation of serology.^[8]^ The manifestation of congenital syphilis may be similar to various congenital infections. For example, congenital syphilis presenting as sepsis is easily misdiagnosed as bacterial sepsis.

The term neonatal sepsis is used to designate a systemic condition that is associated with hemodynamic changes and other clinical manifestations and results in substantial morbidity and mortality.^[9]^ Despite clinical experience in the care of newborns with confirmed or suspected sepsis, there is no consensus on the definition of neonatal sepsis.^[10]^ Neonatal sepsis occurs in utero from either a transplacental or, more commonly, ascending bacteria entering the uterus from the vaginal. It can be the result of infections with bacterial, viral, or fungal microorganisms. Syphilis presenting as sepsis is worthy of consideration even if rare.

In this study, we aimed to identify the benefit of earlier and precisive diagnosis for the infants who suffer congenital syphilis presenting as sepsis.

## 2 Methods

Patients with a documented diagnosis of congenital syphilis presenting as sepsis between 2011 and 2018 were identified from the medical records of The West China Second University Hospital of Sichuan University. Inclusion criteria for this study included

(a) having a confirmed diagnosis of congenital syphilis, based on serology and clinical manifestation,
(b) having a clinical diagnosis of sepsis, the diagnosis of sepsis according to the international guidelines for sepsis diagnostic criteria,^[11]^ and
(c) receiving treatment.

Exclusion criteria included (a) had other congenital infections, for example, HIV, TORCH. All medical records were reviewed, and auxiliary examination results were collected. This information included the main clinical symptoms; the medical records of mother's syphilis treatment; the laboratory test, including the blood routine, the biochemical test, CRP (C-reactive protein), RPR (Realtors Property Resource), TPHA (*Treponema pallidum* particle agglutination assay), CSF (cerebrospinal), X-ray examination, etc. We also collect the recovery and discharge outcome.

In addition, 29 cases who were diagnosed neonatal sepsis that blood culture is positive apart from CS (bacteria or fungus) in the principle of concurrent control is collected. The control group followed the same diagnostic criteria. The same medical records are collected. All the study protocol was approved by the Ethical Committee of West China Second University Hospital.

SPSS statistical software version 22.0 (IBM Corp., Armonk, NY) was used to compare the differences of characteristics between groups. Measurements were presented as means ± standard deviation and were analyzed by using independent sample *t*-test for continuous variables and *χ*^2^ test or Fisher exact test for categorical variables. *P*-values less than .05 were considered statistically significant.

## 3 Results

### 3.1 The general clinical manifestations and laboratory examination results of 29 patients with congenital syphilis presenting sepsis

**Figure 1:**
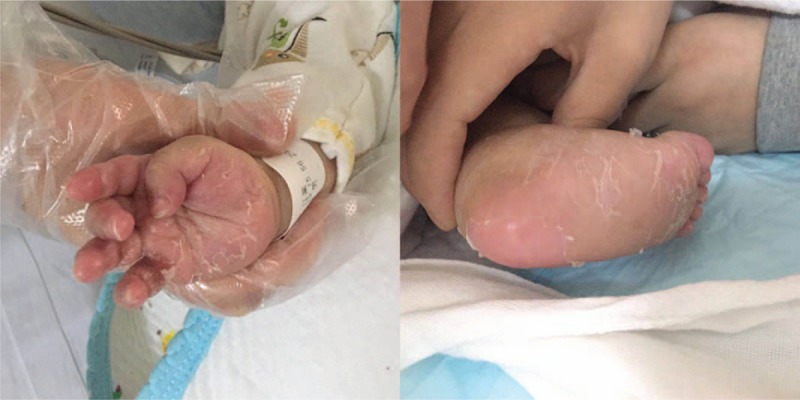
The palms and feet of the patients manifested as large or large pieces of desquamation.

A total of 29 patients involving 20 boys and nine girls between 2011 and 2018 were identified from the medical records of The West China Second University Hospital of Sichuan University in this study Table 1. These patients had been diagnosed clinically with congenital syphilis presenting sepsis. The median age was 14 days (ranging from newborn to 5 months) with 48.2% being younger than 7 days (IQR = 36). All patients had a history of exposure to infected mothers; as a retrospective analysis, it is difficult to know the ratio of adequate blockage treatment to pregnant. The median diagnosis time (designated as the time between onset of symptoms and diagnosis CS) was 2 days (ranging from 0 days to 5 days) in our serial cases. In China, prior to transfusion, patients need routine laboratory pre-transfusion examination tests involving hepatitis B virus surface antigen, hepatitis C virus antibody, the HIV antibody, and the *Treponema pallidum* antibody.^[12]^ Among them, 22 children were accepted transfusion to improve anemia and low platelets, 10 cases have been diagnosed by serology test, rather than 12 of them were diagnosed CS by the pre-transfusion examination. The most common clinical characteristic for the patients was neonatal decreased responsiveness, rash, and abdominal distension. The main clinical manifestations and laboratory examination results of patients are summarized in Table 1. 55.2% of patients presented rash, which is usually scatter or multiple, round, with papules on the periphery, in the mouth, buttocks, palms, and feet. Palmar damage mostly manifested as large or large pieces of desquamation (Fig. 1). The obvious abdominal distension was found in half patients as splenomegaly (55.2%) or/and hepatomegaly (72.4%).

**Table 1:**
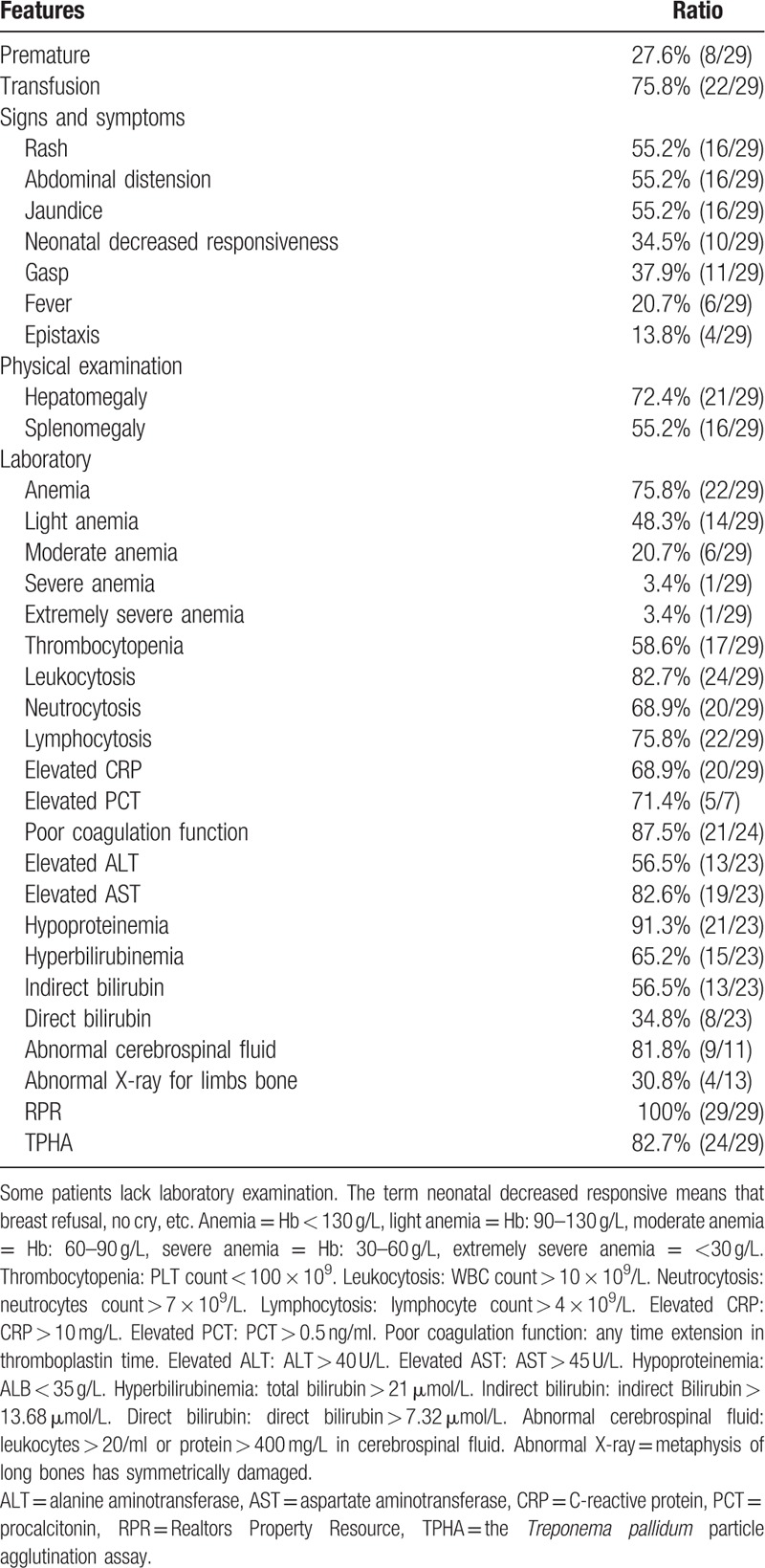
The main clinical manifestations and laboratory examination results of 29 patients with congenital syphilis presenting sepsis.

Abnormalities in hematologic evaluations were found in most patients as 82.1% of patients presented leukocytosis (25.9 ± 14.7 × 10^9^/L), 75% of patients presented anemia (113.3 ± 37.7 g/L), and 57.1% existed thrombocytopenia (110.0 ± 91.7 × 10^9^/L, with a minimum reading of 8 × 10^9^ g/L) in Table 2. 95.2% of patients had hepatic dysfunctions as determined by elevated ALT (118.2 ± 155.8U/L), elevated AST (172.1 ± 216.2U/L), albumin (25.2 ± 4.8 g/L), total bilirubin (79.4 ± 73.4 μmol/L), and indirect bilirubin (45.9 ± 45.8 μmol/L) in Table 2. 20 patients had a poor coagulation function as determined by prolonged APTT and PT. Some unusual manifestations were additionally observed. For example, hemorrhage was manifested as epistaxis and ecchymosis in four patients. No patients were found to suffer from urine or renal dysfunction. In the 13 patients who had the X-ray for limbs bone, four cases reported abnormal; it means these skeletal system involvements. In the 11 patients who had a CSF test, nine cases reported abnormal. As one of the evidence for the diagnosis of congenital syphilis, there are 23 positive TPHA cases. Seven cases of CS were premature infants, someone of them with neonatal respiratory distress syndrome, neonatal cold injury syndrome, etc. 17 cases which combined with pneumonia mostly appeared gasp. In this study, all children studied were exposed to an infected mother though some of the pregnant mothers were accepted intramuscularly procaine penicillin. It is regretful that the details of the blocking in these cases were not found. Except for one patient who died immediately after admission, the other patients received regular penicillin therapy, intravenously, a total dosage of 100–150,000 U/kg dividing into two to three times, usually in 10–15 days. One child was allergic to penicillin and later switched to cefotaxime. In addition to penicillin, these children with congenital syphilis presenting sepsis received appropriate supportive treatments such as fluid resuscitation, respiratory support, vasoactive drugs. The 12 patients who had been progressively worse to MODS did not recover well until their guardians decided to discharge. It is unavailable to acquire the follow-up information after discharge due to lack of the patients’ contact information.

**Table 2:**
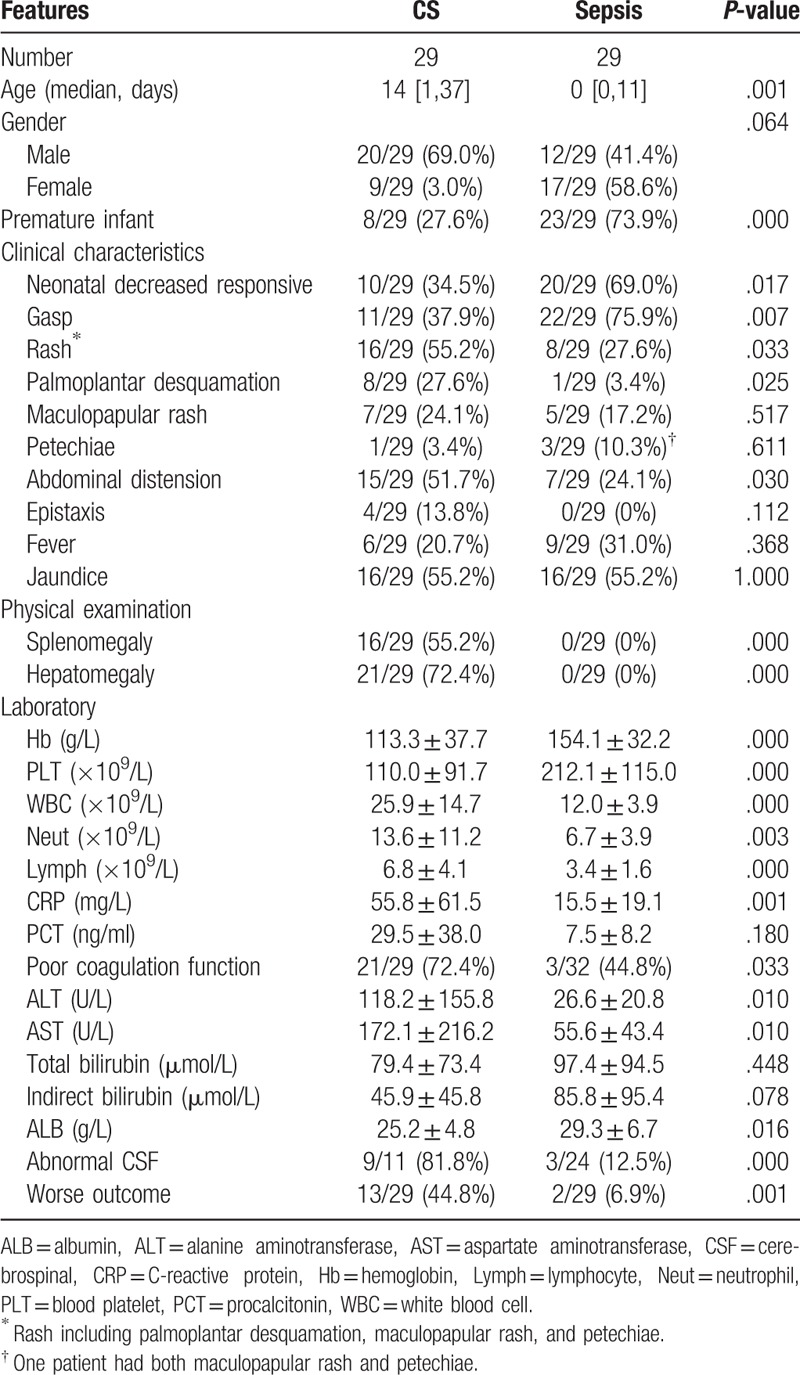
Comparison between the CS group and the neonatal sepsis group.

### 3.2 The difference between congenital syphilis presenting sepsis and neonatal sepsis

Due to the anonymous and the high mortality rate of congenital syphilis presenting sepsis, we look forward to distinguish it between other common sepsis in the early stage Table 2. So, we collected 29 cases who were diagnosed neonatal sepsis apart from CS. These neonatal sepsis patients had positive bacteria or fungus blood culture, for example, *Klebsiella pneumoniae*, *Streptococcus agalactiae*, *Staphylococcus epidermidis*, *Enterococcus faecium*, *Candida*, etc. To investigate the diagnostic indicators between the two groups, different analysis was examined including their general condition, clinical characteristics, and laboratory tests. Statistically significant difference (*P* < .05) is found between the groups on the indicators, that consist of age (14 (1–37) vs. 0 (0–11), *P* = .001), premature (27.6% vs. 73.9%, *P* = .000), neonatal decreased responsiveness (34.5% vs. 69%, *P* = .017), gasp (37.9% vs. 75.9%, *P* = .007), rash (53.6% vs. 25.0%, *P* = .034), epistaxis (13.8% vs. 0%, *P* = .112), abdominal distension (51.7% vs. 24.1%, *P* = .03), splenomegaly (55.2% vs. 0%, *P* = .000), and hepatomegaly (72.4% vs. 6.3%, *P* = 0.000). And, at the aspect of Hb (113.3 ± 37.7 vs. 154.1 ± 32.2, *P* = .000), PLT (110.0 ± 91.7 vs. 212.1 ± 115.0, *P* = .000), WBC (25.9 ± 14.7 vs. 12.0 ± 3.9, *P* = .000), CRP (55.8 ± 61.5 vs. 15.5 ± 19.1, *P* = .001), ALT (118.2 ± 155.8 vs. 26.6 ± 20.8, *P* = .001), AST (172.1 ± 216.2 vs. 55.6 ± 43.4, *P* = 0.001), these differences occurred in the CS group versus the neonatal sepsis group. In the rash, there are three types, including palmoplantar desquamation, maculopapular rash, and petechiae. We found that the palmoplantar desquamation attribute to the main difference in rash between the two groups, 8/29 (27.6%) vs. 1/29 (3.4%), *P* = .025. Finally, it is obvious that the prognosis of children with syphilis is worse (44.8% vs. 6.9%, *P* = .001). Results are presented in Table 2.

### 3.3 Comparison between the different outcomes in the patients with congenital syphilis presenting sepsis

Now, in order to further discuss possible prognostic factors, we try to divide cases into benign outcome and worse outcome as two subgroups for statistical analysis in the CS, the results seen in Table 3. The worse outcome subgroup of patients is significantly younger (age days = 2 vs. 18, *P* = .02) and have more severely impaired liver function, including ALT (149.9 ± 215.4 vs. 93.8 ± 90.7, *P* = .05), AST (222.6 ± 272.2 vs. 131.7 ± 156.9, *P* = .06), ALB (24.3 ± 2.7 vs. 25.9 ± 6.1, *P* = .04), and jaundice (37.5% vs. 76.9%, *P* = .061). Despite no significant difference, non-diagnosed before transfusion seems to be more in the worse outcome subgroup (53.8% vs. 31.3%).

**Table 3:**
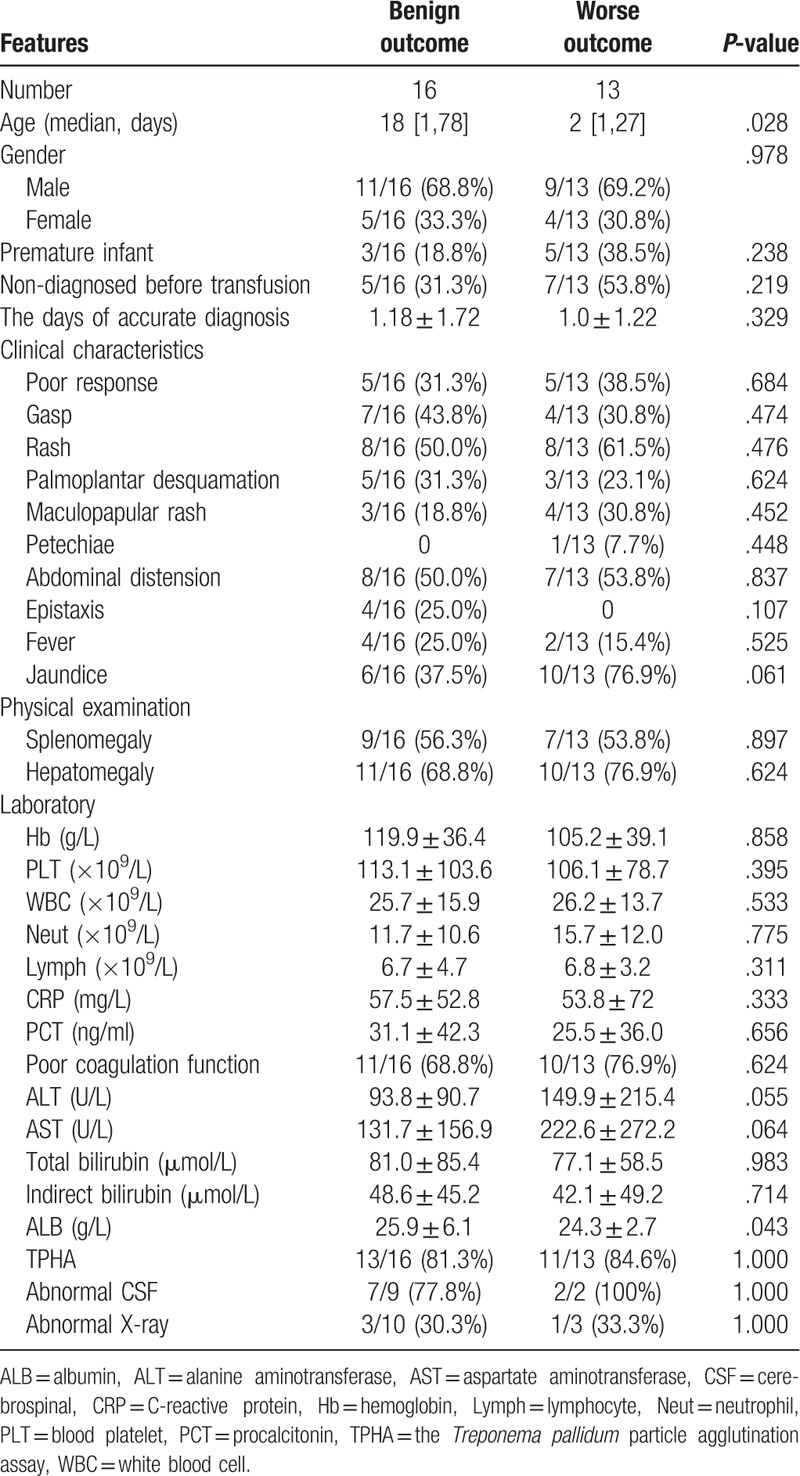
Comparison between the different outcomes in the patients with congenital syphilis presenting sepsis.

## 4 Discussion

CS occurs in an infected mother via mother-to-child transmission. In this study, all children studied were exposed to an infected mother though some of the pregnant mothers were accepted intramuscularly benzathine penicillin. Due to difficulties in diagnosis, asymptomatic infections, and lack of monitoring or reporting systems, the accurate incidence of CS is limited. As is known to all, the incidence of CS is correlated to syphilis. WHO estimates that 5.6 million new cases of syphilis occurred among people aged 15–49 years worldwide in 2012 (1.5 cases per 1000 females and 1.5 per 1000 males).^[6]^ Towards the elimination of CS from 2008 to 2012, it is reported that CS have decreased by 38%.^[13]^ It contributes to WHO and some national guidelines.^[14–16]^ According to these guidelines, syphilis screening is recommended for pregnant women universally, regardless of previous exposure. Treatment of syphilis-seropositive pregnant women is critical for prevention of CS.

Despite this fact, CS should not be ignored for the terrible outcome. Patients with CS presenting sepsis are a group of infants who had a heavy organ dysfunction caused by CS infection. In our study, most of these patients have elevated inflammatory markers and more than one organ dysfunction. Inflammatory marker consisted leukocytosis (82.7%), elevated CRP (68.9%), and elevated PCT (71.4%). Additionally, patients presented abdominal distension (55.2%), splenomegaly (55.2%), hepatomegaly (72.4%), and rash (55.2%). The main pathological changes of CS are fibrosis of organs such as liver, spleen, pancreas, and placenta. The fetal liver becomes larger, and obvious fibrosis and extramedullary hematopoiesis appear. Similar lesions can also occur in the pancreas, spleen, and heart, as well as osteochondritis, skin, etc.^[4]^ Pathologic changes in hepatosplenomegaly and hypersplenism, destruction leading to anemia, low platelet, elevated liver enzymes, hyperbilirubinemia, hypoproteinemia, and coagulation disorders have also been described. In this study, abnormal liver function was reported in 95.2% of patients. In this paper, we first summarize a series of CS presenting as sepsis cases, highlighting the clinical features of these patients, consistent with others reported cases.^[17,18]^ 100% of patients had a positive report in TP, and 82.1% of them are positive to TPHA test. In view of these facts, some indicators, such as hepatosplenomegaly, abnormal liver function, rash, and TP positive etc. seem to help pediatricians to accurately diagnose. But these symptoms and lab test which lack specificity is easily confused with other diseases. So, we compare CS presenting as sepsis with neonatal sepsis.

It is considered that some difference occurs between CS presenting sepsis and neonatal sepsis. The difference between ages could be explained without making age-matching. The neonatal sepsis was chose as our control group, whose high-risk factors consist of premature birth.^[19]^ It is easy to understand why premature infants, gasp, and neonatal decreased responsiveness in the control group are more than the CS group. As the results showed in our study, the CS presenting sepsis should be considered when neonatal sepsis have special characteristics, such as rash, epistaxis, abdominal distension, splenomegaly hepatomegaly, Hb, PLT, ALT, and AST. The higher content of WBC and CRP may suggest the heavier immune response in the CS presenting sepsis. The palmoplantar desquamation attributes to the main difference in rash between two groups, which more commonly occur in the CS.^[20]^

Finally, it is obvious that the prognosis of children with syphilis is worse. According to the comparison between the different outcomes in the CS, the worse outcome subgroup of patients is significantly younger and have more severely impaired liver function. The time for accurate diagnosis seems to be shortened in worse outcome subgroup. The reason for this phenomenon is that the patients are seriously ill and needs a timely blood transfusion to have a pre-transfusion examination. And, it could be seen that early diagnosis and treatment did not achieve better outcome. The most important reason is that the worse outcome of patients contributes to the more severe damage of congenital syphilis infection in the uterus. These patients are more likely to develop CS presenting sepsis in the younger days, due to impaired liver function. In addition, with approximately 30% of pregnancies resulting in fetal death in utero, stillbirth or death shortly after delivery,^[5]^ infants with CS presenting sepsis may be the quite worse condition in the early days than other long-term sequelae. Once condition appears, the intravenous aqueous benzylpenicillin or intramuscular procaine penicillin daily is timely and necessary. When penicillin cannot be used (e.g., due to penicillin allergy), the WHO guideline suggests using doxycycline, ceftriaxone, or azithromycin. According to the strength of RPR, the time of mother treatment, and infection of pregnant mothers, the infants should be closely monitored. If the infants are clinically suspected as CS, penicillin can be used as empirical treatment.^[14]^

Despite the timely treatment, the effect is not particularly useful, especially in those critical patients. So, prevention is more important considering serious consequences. It is obvious that the prognosis of children with syphilis is worse. The worse outcome of patients (44.8%) with CS presenting sepsis contributes to the damage of congenital syphilis infection in the uterus. It is necessary to do syphilis screening recommended for pregnant women and different penicillin treatment plan on screened pregnant women. Once the CS presenting as sepsis occasionally appeared, pediatricians should keep vigilant and diagnose timely in accordance with the specific manifestation showed in our study.

## Acknowledgments

We thank the staff at the Department of Infective Pediatrics, West China Second University Hospital, for their work in collecting the samples.

## Author contributions

YL, YZ, and CW conceptualized and designed this study and applied for funding. YL and YW collected data. YL analyzed the data and wrote the first draft of this paper. All authors revised this paper and approved the final version as submitted.

**Conceptualization:** Yang Liu, Chaomin Wan.

**Data curation:** Yang Liu, Yu Zhu, Yibin Wang.

**Formal analysis:** Yang Liu, Yu Zhu.

**Funding acquisition:** Yang Liu, Chaomin Wan.

**Investigation:** Yang Liu, Yu Zhu.

**Methodology:** Yang Liu, Yu Zhu, Chaomin Wan.

**Project administration:** Yang Liu.

**Resources:** Yang Liu.

**Software:** Yang Liu.

**Supervision:** Yang Liu.

## References

[1] Radolf JD Deka RK Anand A *Treponema pallidum*, the syphilis spirochete: making a living as a stealth pathogen. Nat Rev Microbiol 2016;14:744–59. 27721440 10.1038/nrmicro.2016.141 PMC5106329

[2] Harter C Benirschke K Fetal syphilis in the first trimester. Am J Obstet Gynecol 1976;124:705–11. 56895 10.1016/s0002-9378(16)33340-3

[3] Woods CR Syphilis in children: congenital and acquired. Semin Pediatr Infect Dis 2005;16:245–57. 16210105 10.1053/j.spid.2005.06.005

[4] Kliegman RM Stanton BF St Geme JW Nelson Textbook of Pediatrics. 20^th^ edition. 2015;Philadelphia: Elsevier, 1470–1477.

[5] Newman L Kamb M Hawkes S Global estimates of syphilis in pregnancy and associated adverse outcomes: analysis of multinational antenatal surveillance data. PLoS Med 2013;10:e1001396. 23468598 10.1371/journal.pmed.1001396 PMC3582608

[6] WHO Guideline on Syphilis Screening and Treatment for Pregnant Women. Geneva: World Health Organization; 2017. 29757595

[7] Saloojee H Velaphi S Goga Y The prevention and management of congenital syphilis: an overview and recommendations. Bull World Health Organ 2004;82:424–30. 15356934 PMC2622853

[8] Morshed MG Singh AE Recent trends in the serologic diagnosis of syphilis. Clin Vaccine Immunol 2015;22:137–47. 25428245 10.1128/CVI.00681-14 PMC4308867

[9] Shane AL Stoll BJ Neonatal sepsis: progress towards improved outcomes. J Infect 2014;68 Suppl 1:S24–32. 24140138 10.1016/j.jinf.2013.09.011

[10] Wynn JL Wong HR Shanley TP Time for a neonatal-specific consensus definition for sepsis. Pediatr Crit Care Med 2014;15:523–8. 24751791 10.1097/PCC.0000000000000157 PMC4087075

[11] Dellinger RP Levy MM Rhodes A Surviving Sepsis Campaign: international guidelines for management of severe sepsis and septic shock, 2012. Intensive Care Med 2013;39:165–228. 23361625 10.1007/s00134-012-2769-8 PMC7095153

[12] The National Health Commission of the Republic of China: The technical standard of clinical blood transfusion. 2001. Available at: http://www.nhc.gov.cn/yzygj/s3589/200804/adac19e63a4f49acafab8e0885bf07e1.shtml [accessed March 18, 2019, in Chinese].

[13] Wijesooriya NS Rochat RW Kamb ML Global burden of maternal and congenital syphilis in 2008 and 2012: a health systems modelling study. Lancet Glob Health 2016;4:e525–33. 27443780 10.1016/S2214-109X(16)30135-8 PMC6759483

[14] WHO Guidelines for the Treatment of Treponema pallidum (Syphilis). Geneva: World Health Organization; 2016. 27631046

[15] Centers for Disease Control and Prevention, Syphilis treatment and care. 2015 STD Treatment Guidelines – Syphilis 2015 2015.

[16] Antenatal screening for HIV, hepatitis B, syphilis and rubella susceptibility in the EU/EEA. European Centre for Disease Prevention and Control 2017.

[17] Akahira-Azuma M Kubota M Hosokawa S Republication: Two premature neonates of congenital syphilis with severe clinical manifestations. Trop Med Health 2015;43:165–70. 26543391 10.2149/tmh.2015-11 PMC4593778

[18] Arriagada D Donoso A Cruces P Congenital syphilis: presenting as septic shock alter the neonatal period. Rev Chilena Infectol [in Spanish] 2012;29:558–63. 10.4067/S0716-10182012000600017 23282504

[19] Puopolo KM Benitz WE Zaoutis TE Management of neonates born at ≤34 6/7 weeks’ gestation with suspected or proven early-onset bacterial sepsis. Pediatrics 2018;142: 10.1542/peds.2018-2894 30455342

[20] Lee SH Kim JH Kim SC Early congenital syphilis presenting with vesicobullous eruptions beyond palmoplantar regions. Acta Derm Venereol 2014;94:321–2. 24037177 10.2340/00015555-1703

